# Acknowledgment to Reviewers of *Veterinary Sciences* in 2021

**DOI:** 10.3390/vetsci9020036

**Published:** 2022-01-19

**Authors:** Veterinary Sciences Editorial Office

**Affiliations:** MDPI AG, St. Alban-Anlage 66, 4052 Basel, Switzerland

Rigorous peer-reviews are the basis of high-quality academic publishing. Thanks to the great efforts of our reviewers, *Veterinary Sciences* was able to maintain its standards for the high quality of its published papers. Thanks to the contribution of our reviewers, in 2021, the median time to first decision was 21 days and the median time to publication was 44.5 days. The editors would like to extend their gratitude and recognition to the following reviewers for their precious time and dedication, regardless of whether the papers they reviewed were finally published:
Abbate, Jessica MariaLi, HaoAbugomaa, AmiraLi, NaAchaaban, Mohamed RachidLi, XinweiAcosta, GerardoLiao, Albert T.Addie, DianeLiao, PengAdroher, F. JavierLibeau, GenevièveÁguila, LuisLichtenwalner, AnneAgustinho, Bruna CalvoLierz, MichaelAlberti, ElenaLin, Chen-SiAlberto Díaz-Sánchez, AdrianLinares, Maria BelénAlegria-Moran, RaulLing, ChenAlvarez, ElizabethLiu, HaopingAlves Ferreira, DavidLiu, I-LiAlves, João CarlosLobetti, RemoAmat, SamatLocatelli, ChiaraAmieux, Paul S.Loi, FedericaAmorim, Renee LauferLombardero, MatildeAndrade-Montemayor, Héctor MarioLombardi, PieroAndre, MarcosLopes, Jordana SenaAndré, Marcos RogérioLópez, CeferinoAndré-Fontaine, GenevieveLorella, ManiscalcoAngelou, AthanasiosLouvandini, PatriciaAnna, Lange-ConsiglioLozier, JosephAntanaitis, RamunasLu, Ming-WeiArcangioli, Marie-AnneLuciana, RossiArciszewski, MarcinLuciani, AlessiaArechiga-Ceballos, NidiaLujan, LluisArmando, FedericoLunge, Vagner RicardoArmengol, RamónMacedo Barragán, Rafael JulioArukha, Ananta PrasadMachado, Gisele FabrinoAshraf, Ghulam MdMacrì, FrancescoAsin, JavierMagalhães-Sant’Ana, ManuelAsproni, PietroMair, KerstinAthanasiou, Labrini V.Malik, RichardAttipa, CharalamposMammi, LudovicaAwosile, BabafelaMancianti, FrancescaAzarpajouh, SamanehMancini, LeonardoAzkona, GarikoitzMandigers, Paul J.J.Aznar, Francisco JavierManfredi, MartinaBaccigalupi, LoredanaMansell, PeterBachmann, MartinMarchegiani, AndreaBalasubramanian, BalamuralikrishnanMarioni-Henry, KatiaBalcells, SusanaMarqués, FernandoBalka, GyulaMarques, MarianaBallegeer, MarliesMarruchella, GiuseppeBalzani, AgneseMarschang, Rachel E.Banaszak, MirosławMartin, KarinBandara, Rathnayaka Mudiyanselage Amila SubhashinieMartin, LynnBanks, Scott A.Martín-Cuervo, MaríaBarragan, Adrian A.Martinet, BaptisteBarszcz, MarcinMartinez-Valladares, MariaBarua, AnimeshMartins-Bessa, AnaBasic, AminaMaruccio, LuciannaBastos, ArmandaMasiulis, MariusBatzias, GeorgiosMasucci, MarisaBeasley, ErinMateos, IvánBeck, AlanMatera, Julia MariaBęczkowski, Paweł M.Mathews, Karol AnnBeineke, AndreasMatsuyama, TomomasaBelli, ManuelMauri-Aucejo, Adela R.Bellini, LucaMawson, Peter R.Belluco, SaraMazzotta, ElisaBenazzi, CinziaMcCartney, WilliamBenedetto, MorandiMcCoski, SarahBenmerzouga, ImaanMcGiven, JohnBergmann, HannesMcKeegan, DorothyBertrand, Deputte L.McManus, ConceptaBertzbach, Luca DaniloMcPherson, Andrew S.Best, Caroline M.Medica, PietroBeyi, Ashenafi F.Medina, Gisselle N.Bhaskaran, ShyleshMentaberre, GregorioBiagi, MarcoMerino, Eva Maria PerezBicudo, AlvaroMerle, RoswithaBigliardi, EnricoMesquita, JoãoBillinis, CharalambosMessadi, LiliaBittar, Carla Maris MachadoMetzger, JuliaBlank, RalfMeurer, FábioBloom, PaulMeyer, FlorianBockstahler, BarbaraMeyer, Robert E.Bonelli, PieroMiglio, AriannaBonos, EleftheriosMigliore, SergioBorriello, GiulianoMilanova, AneliyaBorsuk, GrzegorzMiller, David S.Böswald, LindaMiniero, RobertoBouchami, OnsMiranda, LuisaBran, José A.Miranda-de La Lama, Genaro C.Brandt, AdamMissaghi, BayanBrodacki, AntoniMollo, AntonioBrown, Kyle E.Monge, EmmaBrunetti, BarbaraMonti, GustavoBuishand, Floryne O.Montoya-Alonso, J. AlbertoBussalleu, EvaMoore, Matthew D.Bustamante, RocíoMorandi, BenedettoByadgi, Omkar VijayMorris, Kevin N.Byrne, AndrewMotamed, CyrusCaballero, Raúl PérezMucha, JoannaCacheiro-Llaguno, CristinaMucha, MarionCadiergues, Marie ChristineMuíño Otero, RodrigoCaldas, Lucio AyresMunday, JohnCameron, LornaMurakami, MamiCampanile, GiuseppeMurphy, BrianCampion, Deirdre P.Muscher-Banse, Alexandra S.Canadas, AnaMusil, RichardCantalapiedra, AntonioMusk, Gabrielle C.Capela E Silva, FernandoMuttini, AurelioCarnaccini, SilviaMylonakis, Mathios E.Carolina Palacios, JimenezNääs, Irenilza AlencarCaro-Vadillo, AliciaNagata, MasahikoCarral Llamazares, José ManuelNagayasu, EijiCastagnaro, MassimoNamba, ShinichiCastelli, GermanoNarayan, EdwardCerasoli, IlariaNatale, AldaCerro, Ana DelNathanailides, CosmasCésar, Aline Silva MelloNava, GerardoChampetier, AntoineNavas, LuigiChandler, MargeNeerukonda, Naga Venkata SabarinathChang, Geng-RueiNicholson, SandraChang, ShantonNiedźwiedź, ArturCharlotte, MisbachNishida, TakehiroChen, LechuangNissapatorn, VeeranootChen, LiangNocera, Francesca PaolaChen, SeanNock, AdamChen, Shuen-EiNotari, LorellaChidgey, KirstyNovacco, MarilisaChocteau, FlorianNowakiewicz, AnetaChung, Hee-ChunNzelu, Chukwunonso O.Circella, ElenaOcarino De Melo, NataliaClegg, SimonOdemer, RichardCocco, RaffaellaOdongo, DavidColaço, Bruno JorgeOdore, RosangelaCollivignarelli, FrancescoOevermann, AnnaConan, AnneOgawa, ShinichiroConlan, Andrew J. K.Okazaki, KatsunoriConstantino-Casas, FernandoOld, JulieContalbrigo, LauraOliveira, ManuelaCoppock, Robert W.Oliveira, Ronaldo LopesCorda, AndreaOliver, JamesCork, SusanOlkowski, BoguslawCornale, PaoloOrtalda, ChristianCornegliani, LuisaOtranto, DomenicoCosta, Giovanna LucreziaPacífico, CátiaCouto, JoanaPadungtod, PawinCrescenzo, GiuseppePaepe, DominiqueCuevas Ramos, GabrielPallotti, FrancescoDa Silva, Carolina BalaoPalma, MarianaDal Pozzo, FabianaPaltrinieri, SaverioDamiaans, BertPaoletti, BarbaraDaniels, SimonPapach, AnnaDanks, JaninePapadopoulos, GeorgiosD’Ascenzi, CarloParambeth, Joseph CyrusDavidson, Autumn P.Parrilla, InmaculadaDavis, JenniferParvate, AmarDe Araújo Oliveira, Chiara AlbanoParys, MaciejDe Carvalho, Gleidson Giordano PintoPatching, Simon G.De Clercq, DominiquePaterson, MandyDe Lagarde, MaudPavarini, Saulo PetinattiDe Lima Santos, RenatoPawełczyk, OlgaDe Simone, Salvatore GiovanniPayan-Carreira, RitaDe, KuntalPech Cervantes, Andres AlfredoDea-Ayuela, MaríaPeckham, Gabriel D.Debess, Emilio E.Pelagalli, AlessandraDecaro, NicolaPenrith, Mary-LouiseDel Chicca, FrancescaPerego, RobertaDelghandi, MohammadPereira Da Fonseca, IsabelDelhon, GustavoPeretz, AviDelia, LacastaPérez, JoséDennis, Rachel L.Pérez-Martínez, ClaudiaDeschamps, Jack-YvesPerin, PaoloDettori, Maria LuisaPerrucci, StefaniaDeutskens, FabianPetersen, HeidiDevriendt, NausikaaPezzanite, Lynn M.Dhungyel, OmPiantino Ferreira, Antonio JoséDi Bella, SantinaPiccionello, Angela PalumboDi Francesco, Cristina EsmeraldaPierini, AlessioDi Francesco, GabrielePietro, Simona DiDi, RanPietsch, ConstanzeDiakou, AnastasiaPikalo, JuttaDiaz Fernadez, PabloPinello, Kátia CristinaDiaz, DuartePiras, Lisa AdeleDíaz-Godínez, GerardoPisu, Maria CarmelaDidkowska, AnnaPitt, LouiseDiez, RaquelPiva, SilviaDiioia, MarinaPizzorno, MarieD’Incau, MarioPoelaert, KatrienDjokovic, RadojicaPoglayen, GiovanniDobrowolski, PiotrPolak, Mirosław PawełDodds, W. JeanPoli, AlessandroDolka, IzabellaPorporato, MarcoDomańska-Blicharz, KatarzynaPoudel, AnilDomenis, LorenzoPoulsen, Keith P.Dorman, David C.Pourlis, ArisDorny, PierrePrabhu, LakshmiDors, ArkadiuszPrada, JustinaDos Anjos Pires, MariaPugliese, MichelaDos Santos, Regiane RodriguesPyatt, Alison Z.Dotti, SilviaQin, GuyuDroscha, CaseyQueiroga, CristinaDu, XiaoyongQuintas, HelderDu, ZhifengRachael, GrundonDuarte, Marcos EliasRagusa, Maria AntoniettaDuran, CristhianRajeswari, Jithine JayakumarDurham, Amy C.Ramadan, Mohsen M.Edwards, Marten JohnRamirez-Bribiesca, J. EfrenEgizi, AndreaRamírez-Mella, MónicaEhmcke, JensRandhawa, ImtiazEichenberger, Ramon MarcRandle, HayleyEkateriniadou, LoukiaRasschaert, GeertruiElmahallawy, EhabRay, ParthaElolimy, Ahmed A.Razzuoli, ElisabettaEscaffre, OlivierReantaso, MelbaEspí, AlbertoReines, Brandon P.Espunyes, JohanReis, Joana Da CostaEstrada-Angulo, AlfredoRensi, NicolòEvans, Samantha J.M.Reyes-Gomez, EdouardEzquerra Calvo, Luis J.Richards, NgaioFàbrega-Romans, EmmaRiehm, JuliaFan, PeixinRinder, MonikaFarsang, AttilaRiondato, FulvioFatone, GerardoRocha Faustino, Ana IsabelFaustini, MassimoRochfort, KeithFaustino, AnaRodríguez Villanueva, JavierFei, Andrew Chang-YoungRodríguez, Virginia GaminoFerramosca, AlessandraRodriguez-Vargas, Jose ManuelFerrari, RobertaRoghair, Robert D.Ferreira, FernandoRohaim, MohammedFiore, EnricoRoos, EduardFlorín-Christensen, MonicaRossi, LucaFlythe, MichaelRoussel, AnneFonseca-Alves, Carlos EduardoRout, EmilyFowler, HeatherRowland, RaymondFraile, LorenzoRoy, CyrilFraley, Gregory S.Rubbenstroth, DennisFranchini, DeliaRubio San Millan, Luis ÁngelFranciosini, Maria PiaRucinsky, ReneeFranco, Nuno HenriqueSaadeldin, IslamFredes, FernandoSachse, KonradFrey, CarolineSacks, David LawrenceFrossard, Jean-PierreSakai, HirokiFry, Lindsay M.Saleh, Mayra A. D.Fujii, HikaruSaleri, RobertaFyfe, JohnSalimei, ElisabettaGabler, ChristophSaliu, Eva-MariaGalarce, NicolásSalzano, AngelaGalatos, Apostolos D.Sambri, AndreaGalisteo Jr., Andres JimenezSammarco, AlessandroGallucci, AntonellaSánchez, Roberto SánchezGalosi, LivioSanchez-Masian, DanielGarcês, AndreiaSandoval-Castro, CarlosGarcia, MichelleSantarem, NunoGarcía-Barrado, María JoséSantos Junior, Anibal De FreitasGardner, DianneSantos, Andreia A.F.Garigliani, MutienSantos-Jimenez, ZurisadayGarrido, ClaudiaSarli, GiuseppeGärtner, FátimaSatué, KatiuskaGaudreault, Natasha N.Saun, Robert J. VanGeldhof, PeterSavini, LaraGeorge, Kelly A.Schinckel, Allan P.Gerhold, RichardSchoeffmann, GudrunGhisleni, Gabriele C.Schonewille, ThomasGhosh, AnuradhaSchubbert, AntjeGiadinis, Nektarios D.Schwermer, HeinzpeterGiannenas, IliasScollo, AnnalisaGiller, KatrinScott, Nicola Jean AgnesGilmour, MatthewSealy, JoshuaGinevra, BroccaSeifert, JanaGiraud, EmilieSerão, Nick V.Gisder, SebastianSerrapica, FrancescoGoblirsch, Michael J.Sessions-Bresnahan, Dawn R.Gomez, Ernesto A.Shakeri, MajidGómez, MarceloSharma, ArvindGómez-Arrue, JavierSharshov, KirillGomez-Lucia, EsperanzaShephard, Mary N.GONGAL, GyanendraShivley, JakeGonzález, José A.Showler, AllanGopegui, Rafael R.Sid, HichamGórniak, SilvanaSidiropoulou, ErasmiaGradela, AdrianaSigrist, NadjaGraïc, Jean-MarieSilva, EdianeGras, L. (Lapo) MughiniSilvestrini, PaoloGreen, EzekielSimeoni, FrancescoGreenwood, SpencerSimitzis, PanagiotisGreer, AndySimões, João Carlos CaetanoGrimm, HerwigSimões, Margarida PiresGrippi, FrancescaSinha, AmitGrivas, IoannisSinski, Jennifer B.Grzesiak, JakubSitkowska, BeataGuérios, Simone DomitSkampardonis, VassilisGuerri, GiuliaSmets, PascaleGuglielmini, CarloSocha, MagdalenaGuth, AmandaSolano, FranciscoGutierrez-Blanco, EduardoSopena, JoaquínGuzek, DominikaSoroko, MariaHai, RongSotillo, JavierHalliwell, RichardSouza, Alexandre H.Ham, KathleenSøvik, EirikHarkiss, GordonSpada, EvaHarrison, Kelly S.Spadari, AlessandroHawson, Lesley A.Spadavecchia, ClaudiaHayashida, KyokoSpadola, FilippoHedman, HaydenSparks, CathrynHeilen, Laura B.Spinu, MarinaHenrich, EstelleSquire, Sylvia AfriyieHerrera Haro, José GuadalupeStanley, DanaHess, ClaudiaStaton, GarethHeuer, CordStefaniuk-Szmukier, MonikaHidano, ArataSteinhagen, DieterHildebrand, JoannaSteinman, AmirHildebrandt, NicolaiSter, CélineHildreth, Michael B.Stewart, LeslieHill, FraserStraticò, PaolaHirani, RenaStreck, André FelipeHittmair, Katharina M.Stucchi, LucaHoffmann, BerndSutherland, IanHoffmann, Klaus H.Swilam, NohaHofmann, ElisabethSzczepaniak, KlaudiuszHolzhauer, MennoTakehiro, NishidaHsiao, Hui-HuaTakeuchi, HirokiHuang, Guan-JhongTambella, Adolfo MariaHufschmid, JasminTamburro, RobertoHussein, MaythamTanaka, RyouIbañez, Francisco C.Tang, JingjingIglesias Pastrana, CarlosTanui, Collins K.Iglesias, M. José GarcíaTarantola, MartinaInfascelli, FedericoTikhe, Chinmay V.Iniesta Cuerda, MaríaTinelli, AntonellaIsoda, NorikazuTobler, KurtJahns, HanneTomaszewska, EwaJaja, Ishmael FestusTomczuk, KrzysztofJakovcevski, IgorTorres, CristianJamin, MarcTorres-Torres, CarlosJasani, BharatToschi, PaolaJen, PhilipTrabalza-Marinucci, MassimoJerković, IgorTroan, Brigid VictoriaJesus, FatimaTroedsson, Mats H.T.Jewell, Dennis E.Trus, IvanJiang, KangfengTuśnio, AnnaJiang, ShijinTykałowski, BartłomiejJiang, YifengUmair, SalehJohnston, LeeUrimi, DileepJones, Ronald S.Uwiera, RichardJorgensen, MatthewValiakos, GeorgeJung, Dong-InVan Der Weyden, LouiseKafarnik, ChristianeVan Loo, HansKalaitzakis, EmmanouilVannucchi, Camila InfantosiKalle, RaivoVascellari, MartaKamila, BobrekVelasco-Villa, AndrésKang, Byeong-TeckVesce, GiancarloKaramon, JacekViana, Gabriel Da SilvaKarlin, EmilyViegas, CarlosKasman, LauraVigo, DanieleKatayama, KentaroVilar Ares, Maria J.Kato, AtsushiVilla, LucaKatsafadou, AngelikiVillanova, Fabiola ElizabethKatsoulos, Panagiotis D.Vincenti, LeilaKaur, JaspreetVlasov, DmitryKawas, Jorge R.Volta, AntonellaKettunen, PetronellaVyas, DiwakarKhan, Firdous A.Wang, Fun-InKhan, IrfanWarzych-Plejer, EwelinaKim, WootaeWegrzyn, GrzegorzKimbaris, AthanasiosWei, RuifangKizerwetter-Swida, MagdalenaWeina, Peter J.Knight, CameronWestreicher-Kristen, EdwinKochanowski, MaciejWheelhouse, NickKofler, JohannWhitaker, JustineKogut, Michael H.White, Stephen DavidKolbasov, DenisWilliams, EllenKołodziej, PrzemysławWilson, Alphus D.Kondru, NaveenWitkowska-Pilaszewicz, OlgaKowalczyk, PawełWolf, PetraKowalewski, Mariusz PawelWoodward, ElizabethKramer, Mario WolfgangWoźniakowski, GrzegorzKrauze, MagdalenaWysok, BeataKrieg, JochenWysokińska, AnnaKrueger, EddyXie, ShangzheKruitwagen, Hedwig S.Xue, GuangaiKupczyński, RobertYadav, DhananjayLadewig, JanYagound, BorisLadinig, AndreaYen, Chia-HungLai, OlimpiaYin, Hsin-BaiLanglois, DanielYork, DanielLathan, PattyZalewska, MagdalenaLe, Hieu HuuZambonelli, PaoloLeclère, MathildeZamprogno, HeliaLee, DongheonZanetti, Marcus AntonioLee, Wai SumZedda, MarcosLeroux, Louis-PhilippeZerbe, HolmLewis, HelenZeugswetter, FlorianLi, Cong-JunZylinska, Ludmila

